# 1102. Evaluation of Vancomycin Accumulation in Patients with Obesity

**DOI:** 10.1093/ofid/ofab466.1296

**Published:** 2021-12-04

**Authors:** Maha Assadoon, Jeffrey C Pearson, David W Kubiak, Mary P Kovacevic, Brandon Dionne

**Affiliations:** Brigham and Women’s Hospital, Boston, Massachusetts

## Abstract

**Background:**

Current vancomycin guidelines recommend using actual body weight for dosing. However, in patients with obesity, this may result in lower initial vancomycin concentrations that can accumulate with continued doses due to differences in volume of distribution. The objective of this study is to evaluate the incidence of vancomycin accumulation in patients with obesity and identify potential factors associated with accumulation.

**Methods:**

This is a single-center, retrospective, observational study at a tertiary academic medical center. Adult patients with a BMI ≥ 30 kg/m^2^ and with ≥ 2 vancomycin serum trough concentrations within the same encounter in 2019 were screened. Patients were excluded if they were pregnant, had unstable renal function or severe renal impairment, received < 3 doses before a concentration was drawn, or had inconsistent dosing prior to a concentration draw. Linear kinetics were used to correct for differences in timing of concentration or dose changes. The major endpoint was the incidence of vancomycin accumulation, defined as a 20% increase in trough concentration between the first and any subsequent trough concentrations within the first 10 days of therapy. Minor endpoints included the percentage of supratherapeutic concentrations and the incidence of acute kidney injury (AKI). Descriptive statistics were used to evaluate endpoints and multivariable logistic regression was used to evaluate factors associated with accumulation.

**Results:**

We screened 543 patients, and 162 were included in our analysis. The median age was 56.5 years (interquartile range [IQR] 43 - 65.3), and 62.3% were male. The median weight was 112.7 kg (IQR 99.8 - 122.6) and the median BMI was 36.8 kg/m^2^ (IQR 33.1 - 41). The median total daily vancomycin dose at initiation was 28.7 mg/kg/day (IQR 25.4 - 31.2). Vancomycin accumulation occurred in 99 patients (61.1%) within the first 10 days of therapy and AKI occurred in 21 patients (14.9%). No factors studied, including age, gender, obesity class, initial dose, SCr, or frequency were associated with accumulation.

**Conclusion:**

Most patients with obesity experienced vancomycin accumulation within the first 10 days of therapy. Providers should be cautious when assessing a vancomycin concentration early in the treatment course.

**Disclosures:**

**All Authors**: No reported disclosures

